# Remodulation Effect of *Elateriospermum tapos* Yoghurt on Metabolic Profile of Maternal Obesity Induced Cognitive Dysfunction and Anxiety-like Behavior in Female Offspring—An In Vivo Trial on Sprague Dawley Rats

**DOI:** 10.3390/foods12081613

**Published:** 2023-04-11

**Authors:** Ruth Naomi, Rusydatul Nabila Mahmad Rusli, Soo Huat Teoh, Hasnah Bahari, Zainul Amiruddin Zakaria

**Affiliations:** 1 Borneo Research on Algesia, Inflammation and Neurodegeneration (BRAIN) Group, Faculty of Medicine and Health Sciences, Sabah Universiti Malaysia, Kota Kinabalu 88400, Malaysia; gs60018@student.upm.edu.my; 2 Department of Human Anatomy, Faculty of Medicine and Health Sciences, Universiti Putra Malaysia, Serdang 43400, Malaysia; 3 Advanced Medical and Dental Institute, Universiti Sains Malaysia, Penang 13200, Malaysia

**Keywords:** yoghurt, transgenerational obesity, anxiety, cognition, metabolic profile

## Abstract

Pre-pregnancy weight gain induces dysregulation in the metabolic profile of the offspring, thereby serving as a key factor for cognitive decline and anxiety status in the offspring. However, early probiotic supplementation during the gestational period is linked with improved metabolic health. At the same time, a natural plant known as *Elateriospermum tapos* (*E. tapos*) is proven to improve cognition and modulate the stress hormone due to its high concentration of flavonoids. However, the effects of medicinal plant integrated probiotics in F1 generations warrants further investigation. Thus, this study aimed to study the effect of *E. tapos* yoghurt on the maternal obesity induced cognitive dysfunction and anxiety in female offspring. In this study, female Sprague Dawley rats were fed with normal chow (*n* = 8) or high fat diet (*n* = 40) across pre-pregnancy, gestation, and weaning. The treatment with different concentrations of *E. tapos* yoghurt (5, 50, and 500 mg/kg/day) were initiated in the obese dams upon post coitum day 0 up to postnatal day 21 (PND 21). Female offspring were weaned on PND 21 and body mass index, waist circumference, lee index, behavior, metabolic parameter, and antioxidant status were analyzed. The result shows that the female offspring of the 500 mg/kg *E. tapos* yoghurt supplemented group shows a decreased level of insulin, fasting blood glucose, cholesterol, triglycerides, LDL, low fat tissue mass with a high level of HDL, and an increased level of antioxidant status in the hypothalamus. The behavioral assessment proves that the female offspring of the 500 mg/kg *E. tapos* yoghurt supplemented group exhibits a high recognition index on novel object/place with low anxiety-like behavior in an open field test. In conclusion, our data signify the beneficial effect of early intervention in obese dams on the transgenerational impact on female offspring’s metabolic profile, cognitive performance, and anxiety-like behavior.

## 1. Introduction

Maternal obesity has raised the concern of public health as it is currently shows an increasing trend similar to the obesity prevalence rate globally. A study based on panel data model on global maternal obesity shows that there were more than 14 million pregnant obese women worldwide in the year of 2014 [1]. Maternal obesity is an intergenerational vicious cycle [2] and it is a key factor for cognitive decline and anxiety-like behavior in offspring [3]. There is also a strong association between maternal obesity and metabolic changes in the offspring. In fact, maternal obesity is one of the primary causes of a high level of adiposity, increased body mass index (BMI), and dysregulated lipid profile in the child [4]. In this context, a maternal obese woman tends to supply overnutrition to the fetus, which may induce changes in the placental gene expression. This may alter some of the major signaling to the fetus and increase the energy intake during the postnatal period. This condition may worsen in the case of excessive or persistent intake of a high fat diet (HFD) during pregnancy [5]. It is because HFD intake could impair the appetite regulator pathway in the hypothalamus of the fetus, which often is linked with alteration in the leptin and insulin signaling pathway [6]. These permanent damages involving neuroendocrine signaling may suppress the satiety, increase appetite, and adiposity concentration in the growing fetus. As a consequence, the child is at high risk of developing childhood obesity [7]. It has been proven that maternal obesity is one of the primary causes of epigenetic modifications in the offspring that influences the body composition of the offspring, which poses a long-term complication in the child [8]. Along with this, HFD intake during the gestational period could stimulates the formation of proinflammatory cytokines, causing the lipolysis of the adipose tissue. Resultantly, the level of free fatty acids will rise in the plasma of both the maternal obese subject and fetus [9]. On the flip side, consumption of HFD will stimulate the formation of reactive oxygen species (ROS) in the mitochondrial DNA, thereby inducing DNA mutations of the brain. In such case, the endogenous antioxidants will be suppressed. An increased level of ROS will interfere with synaptic transmission of the neurons, causing neuroinflammation, and eventually into neuronal dysfunction [10]. This may manifest as poor cognitive performance in the child. Alike, the child may exhibit anxiety-like behavior [11]. It has been hypothesized that females are more prone to develop later life cognitive dysfunction compared to males [12]. Therefore, it requires urgent attention to curb maternal obesity-linked complications in the female offspring. 

Thus, our recent discovery demonstrates that probiotic intake during the gestational period modulates the metabolic profile of pregnant obese women and the offspring [13]. Concurrently, we found that a natural tropical plant that is commonly available in Southeast Asia, known as *Elateriospermum tapos* (*E. tapos*), comprises of high levels of different types of flavonoids, which are proven to improve endogenous antioxidants in the brain [14]. Besides, *E. tapos* seed is known for its high level of protein, unsaturated fatty acids, oleic acid, linoleic acid, omega-3 essential fatty acid [15], saponins, sterols, tannins, and phenols [14] which possess numerous beneficial effects to human health. Most of the identified compounds in *E. tapos* seed have a molecular weight of less than 600 daltons, implying its capacity to cross the placenta and blood brain barrier to exhibit its effects. In addition, preliminary studies on *E. tapos* extract have shown that it possess the ability to prevent fat absorption by inhibiting the action of pancreatic lipase [16]. A follow-up study provides further evidence that *E. tapos* extract could inhibit the activity of α-amylase, α-glucosidase, and lipoprotein lipase [17], demonstrating the ability of *E. tapos* seed as an effective anti-obesity agent. In fact, *E. tapos* seed is scientifically proven to modulate the stress hormone in F1 generation of obese dams [18]. Hence, we theorize that integrating *E. tapos* into a probiotic could be more effective in inhibiting transgenerational effects of maternal obesity. Thus, this study aimed to study the effect of *E. tapos* yoghurt on the maternal obesity induced cognitive dysfunction and anxiety in female offspring. Accordingly, the outcome from this study could provide new insight into the beneficial effect of novel formulated first medicinal plant integrated yoghurt (*E. tapos* yoghurt) on modulating female offspring’s metabolic profile, cognitive performance, and anxiety-like behavior.

## 2. Materials and Methods

### 2.1. Collection, Confirmation, and Extraction of E. tapos Seed

*Elateriospermum tapos* (*E. tapos*) was supplied by the Research Centre of Forest Research Institute of Malaysia and was sent to Herbarium Biodiversity Unit at Universiti Putra Malaysia (UPM) for confirmation under the voucher code of UPM SK 3154/17. Upon confirmation, the extraction of *E. tapos* seed was done using 2 L of 95% of ethanol. The seed was soaked in ethanol for 7 days, filtered using rotary evaporator. The crude extract was mixed with maltodextrin powder and oven dried overnight until the powder form of *E. tapos* was obtained. The *E. tapos* powder was then refrigerated at −20 °C until further usage [19]. 

### 2.2. Formulation of E. tapos Yoghurt

Plain yoghurt was formulated as an initial method before being integrated with *E. tapos* powder. For this, 100 mL of full cream supplied by Dutch Lady Purefarm UHT was boiled for 30 min, cooled down at room temperature until the temperature dropped to 40–45 °C. Starter culture obtained from a New England cheesemaking supplier was then added to the pre-boiled milk and incubated in a yoghurt maker (Pensonic PYM-700) overnight. The following day, the yoghurt was refrigerator for 12 h and 2 g of *E. tapos* powder was added to the yoghurt [20]. 

### 2.3. Animals and Diet 

All animal protocols were conducted in accordance with the guideline approved by the Institutional Animal Care and Use Committee (IACUC), UPM (UPM/IACUC/AUP-R025/2022). The ethics was approved by IACUC, UPM on 24 June 2022. In this, female rats (*n* = 48) were obtained from Saintifik Entreprise, and were housed in an animal facility in UPM, with a controlled 12 h light and dark cycle (22 ± 3 °C). All rats were fed with standard rat pellet (Gold Coin Feedmills (M) Sdn Bhd, Selangor, Malaysia) with free access to water (ad libitum) [21]. After one week of acclimatization period, rats (*n* = 40) were fed a high fat diet (HFD) pellet comprising of 43% of carbohydrates, 17% of protein, and 40% of fats for 16 weeks to induce obesity [22]. The rest of the rats (*n* = 8) were fed a standard rat pellet with ad libitum. The obesity was confirmed by measuring the significant increase of 13% of mean value compared to the control group [23]. 

### 2.4. Mating, Gestation, and Lactation 

Upon successful obesity induction, all female rats were mated with standard rat pellet fed male Sprague Dawley rats by co-housing 2 female rats with one male rat for 5–7 days. During the mating period, the female rats were checked for pregnancy daily at 8 am by manual palpation followed by vaginal smears. The presence of sperms were observed using a microscope (KF2; Carl Zeiss, Hamburg, Germany) and the first day of sperm detection was recorded as post coitum day 0 [24]. Upon confirmation of pregnancy, females were housed in a single cage and treatment with different *E. tapos* yoghurt concentrations were given through oral administration to the obese dams up to the postnatal day (PND 21). The litters were adjusted to at least 8 female offspring per dams prior to the treatment. The treatment groups are as follows: normal chow and saline (NS), HFD and saline (HS), HFD and yoghurt (HY), HFD and 5 mg/kg of *E. tapos* yoghurt (HYT5), HFD and 50 mg/kg of *E. tapos* yoghurt (HYT50), and HFD and 500 mg/kg of *E. tapos* yoghurt (HYT500).

### 2.5. Weaning and Anthropometrical Determinations

On PND 21, the body length, body weight, and waist circumference of the female offspring were recorded. The BMI and lee index were calculated using the formula below. 

(a) Body mass index (BMI) = body weight (g)/length^2^ (cm^2^), in which the BMI > 0.687 g/cm^2^ was considered as obese [25]. 

(b) Lee index = cube root of body weight (g)/length (cm), in which the Lee index value > 310 g was considered obese [26]. 

### 2.6. Open Field Test 

The anxiety-like behavior of female offspring was accessed using an open field test (OFT) during the light phase of the illumination cycle. In this, a PVC box, measuring 80 cm width × 80 cm length with a 50 cm of height, provided by Muromachi Kikai Co., Tokyo, Japan, was used. The box was divided into two regions by an imaginary line (central & outer region) drawn in the recording software (ANY-maze™ Video Tracking System, Stoelting Co., Wood Dale, IL, USA). Upon setting up the apparatus for OFT, the rats were dropped from one particular area in the box for 5 min. The total distance traveled (m) in the central region, time of exploration in the central and outer region, were recorded using the recording software [27]. 

### 2.7. Novel Object and Place Recognition Test 

For the novel object recognition test (NORT) and place recognition test (PRT), a PVC box, measuring 80 cm width × 80 cm length with a 50 cm of height, provided by Muromachi Kikai Co., Tokyo, Japan, was used. For NORT and PRT, the rats were allowed to explore the empty box for 10 min for 2 days continuously. On the 3rd day, during the trial phase, the rats were allowed to explore two identical objects (mugs) for 5 min, followed by another 5 min of a retention phase. During the test phase, a new object was replaced with one of the mugs and the explorative time at the novel object by the rats was recorded using the ANY-maze™ Video Tracking System. A similar protocol was used for PRT, however, in PRT, two identical objects were placed in one selected location (north and south) initially. During the test phase, one of the identical objects was transferred to the new location (west). In PRT, the explorative time at the new location by the rats was recorded using the ANY-maze™ Video Tracking System [28]. 

### 2.8. Metabolic Profile

On PND 21, all female offspring were fasted for 12 h prior to the fasting blood glucose (FBG) analysis. For this, the tail of the rats was pricked and the blood was sucked using a glucose strip, and the reading was recorded using a glucometer (Glucocard™ 01-mini, Arkray Factory, Inc., Kyoto, Japan) [29]. The rats were then sacrificed using a carbon dioxide overdose and blood was collected and centrifuged for 5 min at 3500 rpm to obtain the plasma. The plasma was then used to analyze the lipid profile using a diagnostic reagent test kit using Hitachi Automatic Analyzer 902 (Tokyo, Japan) [30] and insulin level using commercial rat insulin ELISA kits supplied by Shibayagi Co., Ltd., Gunma, Japan. The retroperitoneal fat (RpWAT), visceral fat, brown adipose tissue (BAT), and gonadal fat were weighed and recorded.

### 2.9. Antioxidants Status 

**Figure 1:**
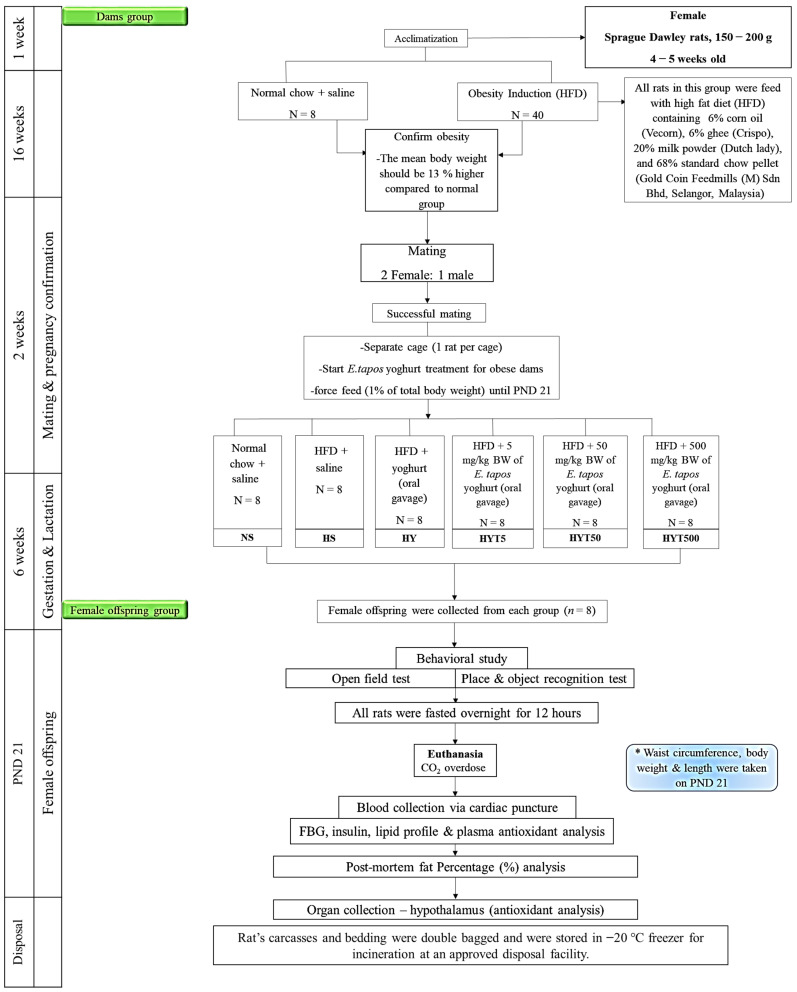
The experimental design.

The hypothalamus was minced into small pieces and diluted in 1:15 of phosphate buffer saline prior to homogenization (Omni TH, Omni International, Kennesaw, GA, USA). The samples were then sonicated 3 times continuously using an ultrasonic cell disrupter (UP 400S, Hielscher, Teltow, Germany) in which each cycle lasted for 20 s. The final samples were collected, centrifuged for 20 min at 5000 rpm, and the level of the ferric reducing ability of plasma (FRAP) and glutathione (GSH) were measured using double-antibody sandwich enzyme-linked immunosorbent assay ELISA kits by Cayman Chemical Company, Ann Arbor, MI, USA [31]. Meanwhile, the serum concentration of FRAP and GSH were analyzed using the same kit. Figure 1 describes the study design of this experimental study. 

### 2.10. Statistical Analysis 

SPSS version 27.0 (Armonk, NY, USA) was used to analyze data obtained from this study. The normality test was completed on all data to ensure normal distribution. Two-way ANOVA was used to compare between all 6 groups for BMI, lee index, waist circumference, recognition index, antioxidant status, and metabolic parameter. This was followed by post hoc to determine the significance among different groups through Tukey HSD correction.

## 3. Results

### 3.1. Anthropometrical Determinations

#### 3.1.1. *E. tapos* Yoghurt Reduces Body Mass Index of Female Offspring 

**Figure 2:**
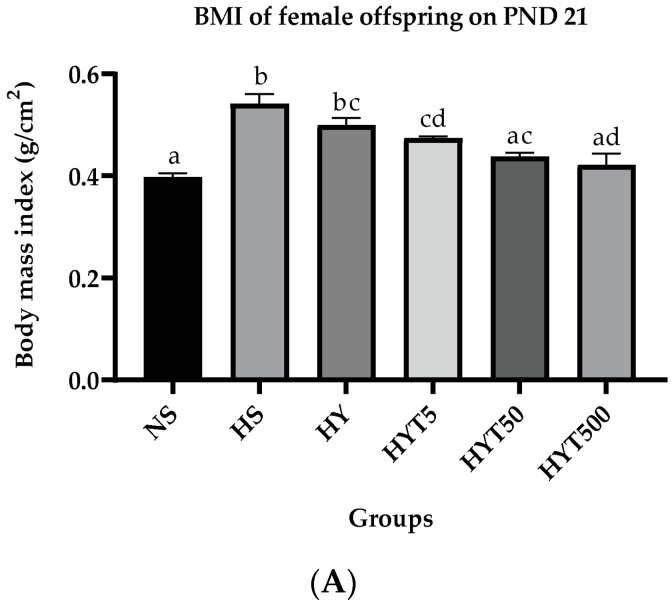

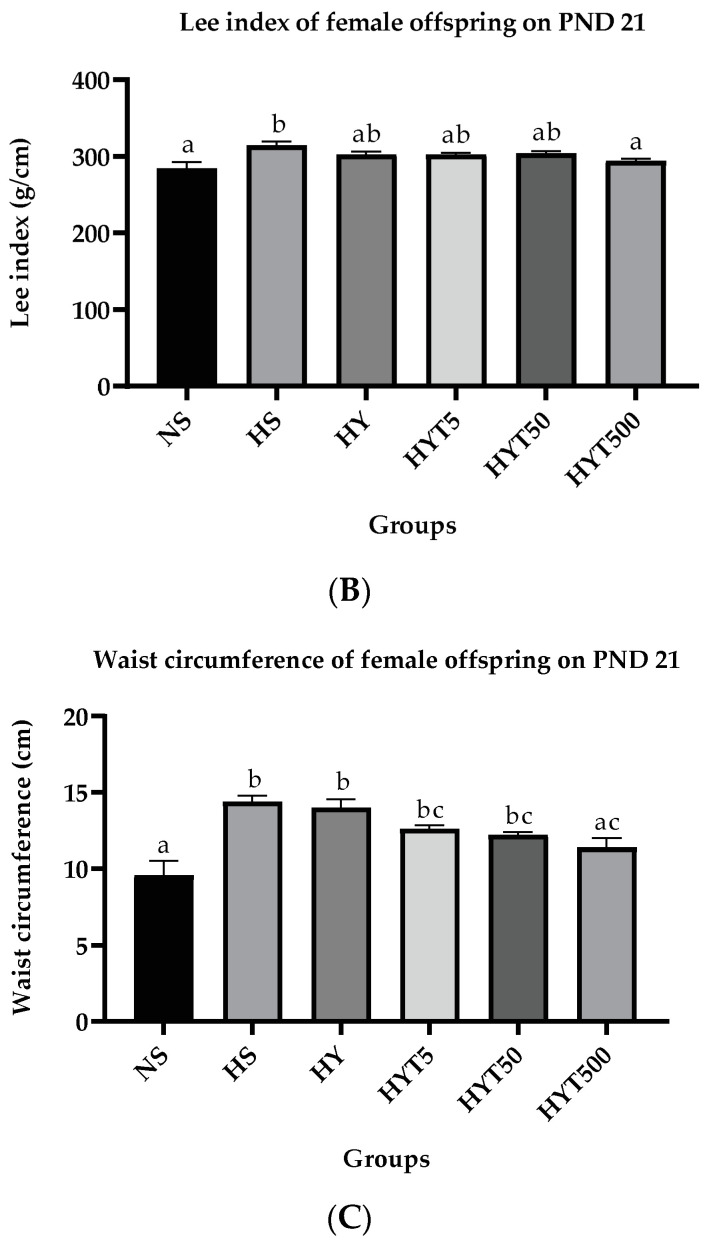
(**A**). Body mass index of female offspring on PND 21. (**B**). Lee index of female offspring on PND 21. (**C**). Waist circumference of female offspring on PND 21. NS: normal chow and saline; HS: HFD and saline; HY: HFD and yoghurt; HYT5: HFD and 5 mg/kg of *E. tapos* yoghurt; HYT50: HFD and 50 mg/kg of *E. tapos* yoghurt; HYT500: HFD and 500 mg/kg of *E. tapos* yoghurt. Values are expressed as mean ± SEM. Different letters indicate a significant difference at *p* < 0.05.

Figure 2A shows the body mass index of female offspring on PND 21. The data show that the BMI of female offspring in HS, HY, and HYT5 groups is significantly high (*p* < 0.05) compared to NS. The BMI of HYT5, HYT50, and HYT500 is significantly low (*p* < 0.05) compared to HS. There is no significant difference (*p* > 0.05) in BMI between HYT5 and HYT50 compared to HY. The BMI of HYT500 is significantly low (*p* < 0.05) compared to HS, HY, HYT5, and HYT50 with a mean BMI value similar to NS.

#### 3.1.2. *E. tapos* Yoghurt Decreases Lee Index of Female Offspring

Figure 2B shows the lee index of female offspring on PND 21. The data show that the lee index of female offspring in HS is significantly high (*p* < 0.05) with a mean value > 310 g compared to NS. There is no significant difference (*p* > 0.05) in lee index between HY, HYT5, and HYT50 compared to HS and NS. Meanwhile, the lee index of HYT500 is significantly low (*p* < 0.05) compared to HS, with a similar mean value as in NS.

#### 3.1.3. *E. tapos* Yoghurt Decreases Waist Circumference in Female Offspring

Figure 2C shows the waist circumference of female offspring on PND 21. The data demonstrate that the waist circumference of female offspring in HS, HY, HYT5, and HYT50 groups is significantly high (*p* < 0.05) compared to NS. There is no significant difference (*p* > 0.05) in waist circumference between HYT5 and HYT50 compared to HY and HS. Meanwhile, the waist circumference of HYT500 is significantly low (*p* < 0.05) compared to HS and HY, while there is no significant difference (*p* > 0.05) of waist circumference among HYT500, HYT50, and HYT5. However, the mean value of HYT500 for waist circumference is comparable to NS.

#### 3.1.4. *E. tapos* Yoghurt Suppress Anxiety-like Behavior in Female Offspring

**Figure 3:**
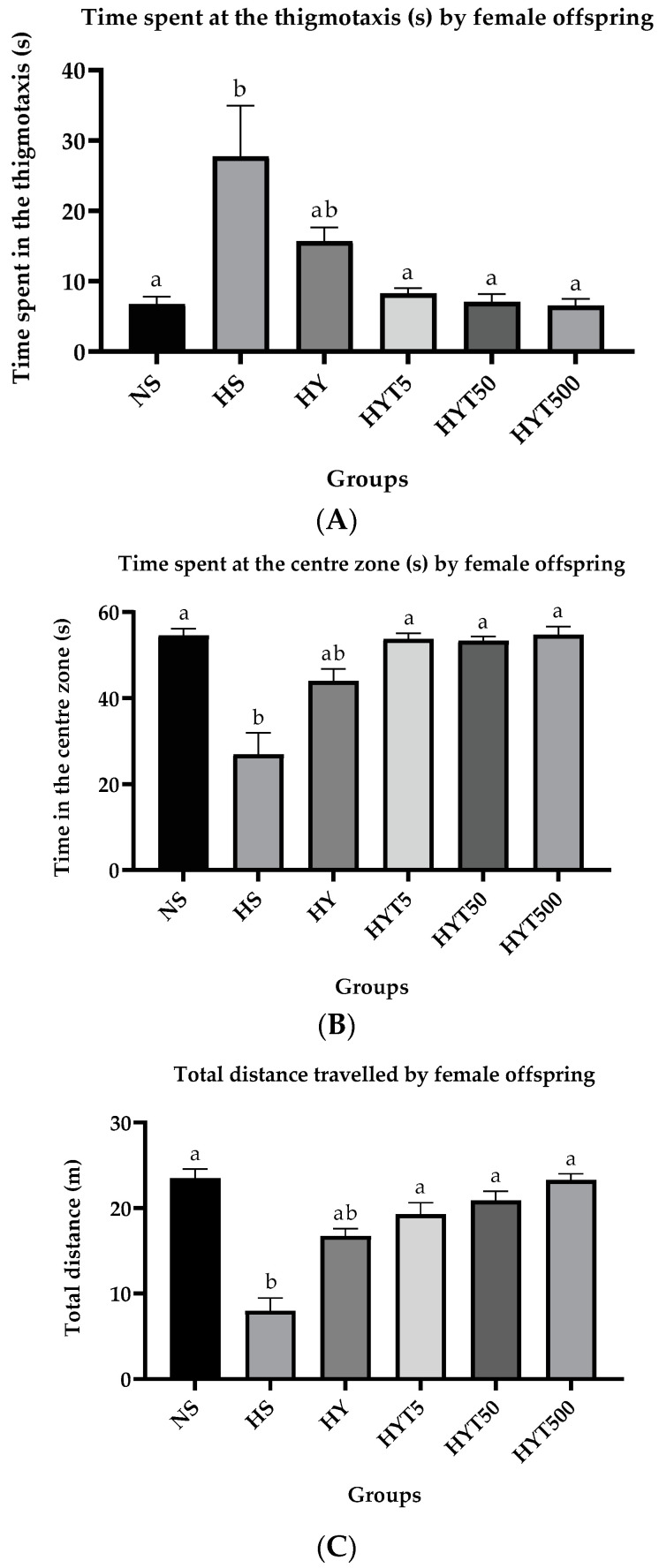
(**A**). Time spent at the thigmotaxis by the female offspring in open field test. (**B**). Time spent at the central zone by the female offspring in open field test. (**C**). The total distance traveled by the female offspring in the central zone of open field test. NS: normal chow and saline; HS: HFD and saline; HY: HFD and yoghurt; HYT5: HFD and 5 mg/kg of *E. tapos* yoghurt; HYT50: HFD and 50 mg/kg of *E. tapos* yoghurt; HYT500: HFD and 500 mg/kg of *E. tapos* yoghurt. Values are expressed as mean ± SEM. Different letters indicate a significant difference at *p* < 0.05.

Figure 3A–C shows the data obtained from the open field test for female offspring on PND 21. As shown in Figure 3A, the female offspring of HS spend significantly high (*p* < 0.05) duration of time near the wall (thigmotactic area) compared to female offspring of NS. There is no significant difference (*p* > 0.05) in the time spent at the thigmotactic region of HY compared to HS and NS. Female offspring of HYT5, HYT50, and HYT500 spend significantly low (*p* < 0.05) duration in the thigmotactic area compared to female offspring of HS. The mean value of HYT5, HYT50, and HYT500 for the explorative duration in the thigmotaxis is comparable to NS. The data in Figure 3B show that the female offspring of HS spend significantly low (*p* < 0.05) duration of time at the central region compared to female offspring of NS. There is no significant difference (*p* > 0.05) in the time spent at the central area of HY compared to HS and NS. Female offspring of HYT5, HYT50, and HYT500 spend significantly high (*p* < 0.05) duration in the central region compared to female offspring of HS. The mean value of HYT5, HYT50, and HYT500 for the explorative duration in the central region is similar to NS. Meanwhile, the data from Figure 3C demonstrate that the total distance traveled by female offspring in the central zone of HS is significantly low (*p* < 0.05) compared to female offspring of NS. There is no significant difference (*p* > 0.05) in the total distance traveled HY in the central zone compared to HS and NS. The total distance traveled female offspring of HYT5, HYT50, and HYT500 in the central zone is significantly high (*p* < 0.05) compared to female offspring of HS. The mean value of HYT5, HYT50, and HYT500 for the total distance traveled in the central zone shows no significant difference (*p* > 0.05) compared to NS, while the mean value of HYT500 is identical to the NS group.

#### 3.1.5. *E. tapos* Yoghurt Increases the Explorative Behavior in Female Offspring

**Figure 4:**
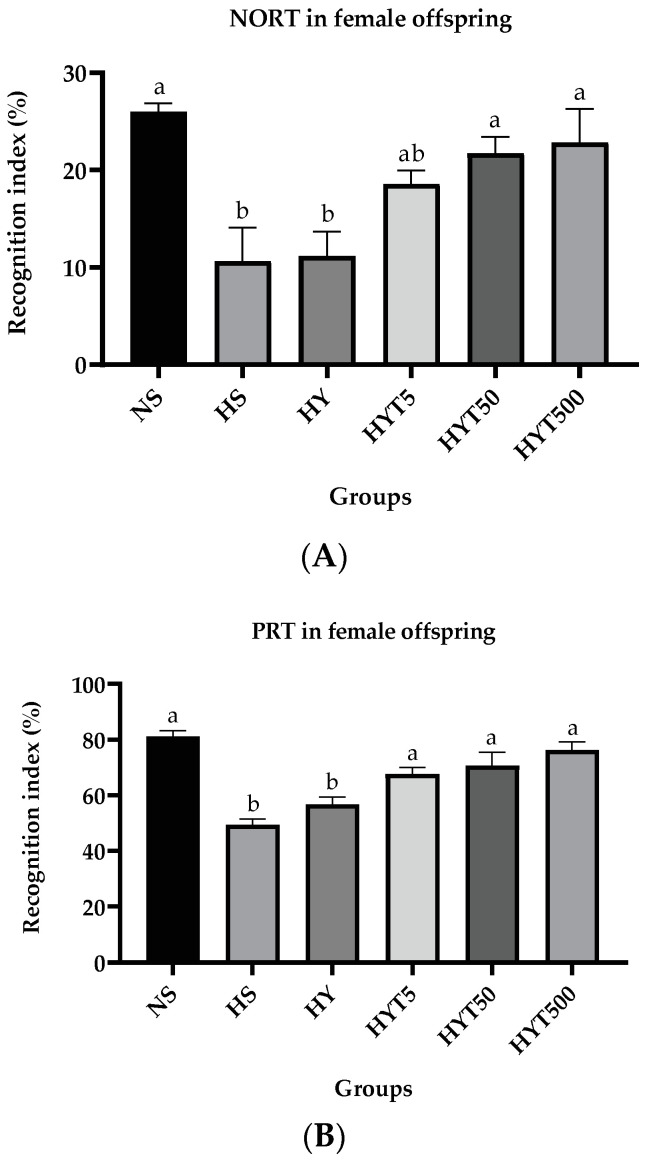
(**A**). Recognition index (%) of female offspring in NORT on PND 21. (**B**). Recognition index (%) of female offspring in PRT on PND 21. NS: normal chow and saline; HS: HFD and saline; HY: HFD and yoghurt; HYT5: HFD and 5 mg/kg of *E. tapos* yoghurt; HYT50: HFD and 50 mg/kg of *E. tapos* yoghurt; HYT500: HFD and 500 mg/kg of *E. tapos* yoghurt. Values are expressed as mean ± SEM. Different letters indicate a significant difference at *p* < 0.05.

Figure 4A,B demonstrates the recognition index of female offspring on NORT and PRT. As shown in Figure 4A, the recognition index of female offspring in HS and HY is significantly low (*p* < 0.05) compared to NS in NORT. There is no significant difference (*p* > 0.05) in the recognition index of HYT5 compared to HS and NS in NORT. Female offspring of HYT50 and HYT500 demonstrate a significantly high (*p* < 0.05) level of the recognition index recognizing novel objects in NORT compared to female offspring of HS. Thus, as shown in Figure 4A, there is no significant difference in recognition index between HYT50, HYT500, and NS in NORT. As shown in Figure 4B, the recognition index of female offspring in HS and HY is significantly low (*p* < 0.05) compared to NS in PRT. Female offspring of HYT5, HYT50, and HYT500 demonstrate a significantly high (*p* < 0.05) level of the recognition index in PRT compared to female offspring of HS and HY. Thus, as shown in Figure 4B, there is no significant difference in the recognition index between HYT5, HYT50, HYT500, and NS in NORT.

#### 3.1.6. *E. tapos* Yoghurt Decreases Fasting Blood Glucose Level in Female Offspring

**Figure 5:**
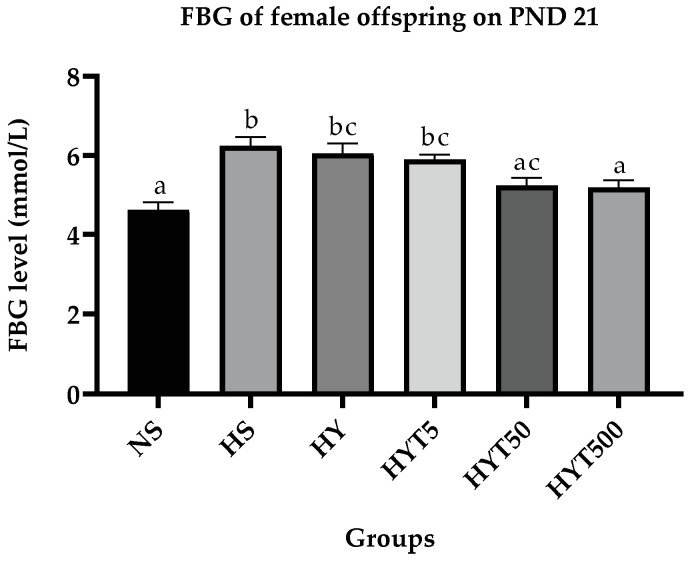
Level of fasting blood glucose in female offspring on PND 21. NS: normal chow and saline; HS: HFD and saline; HY: HFD and yoghurt; HYT5: HFD and 5 mg/kg of *E. tapos* yoghurt; HYT50: HFD and 50 mg/kg of *E. tapos* yoghurt; HYT500: HFD and 500 mg/kg of *E. tapos* yoghurt. Values are expressed as mean ± SEM. Different letters indicate a significant difference at *p* < 0.05.

Figure 5 shows the FBG of female offspring on PND 21. Based on the data, the FBG level of female offspring in HS, HY, and HYT5 is significantly high (*p* < 0.05) compared to NS. There is no significant difference (*p* > 0.05) in the FBG level between HY, HYT5, and HYT50. The FBG level of HYT50 and HYT500 is significantly low (*p* < 0.05) compared to HS. However, the mean value of HYT500 for the FBG level is comparable to NS.

#### 3.1.7. *E. tapos* Yoghurt Modulates Lipid Profile in Female Offspring

**Figure 6:**
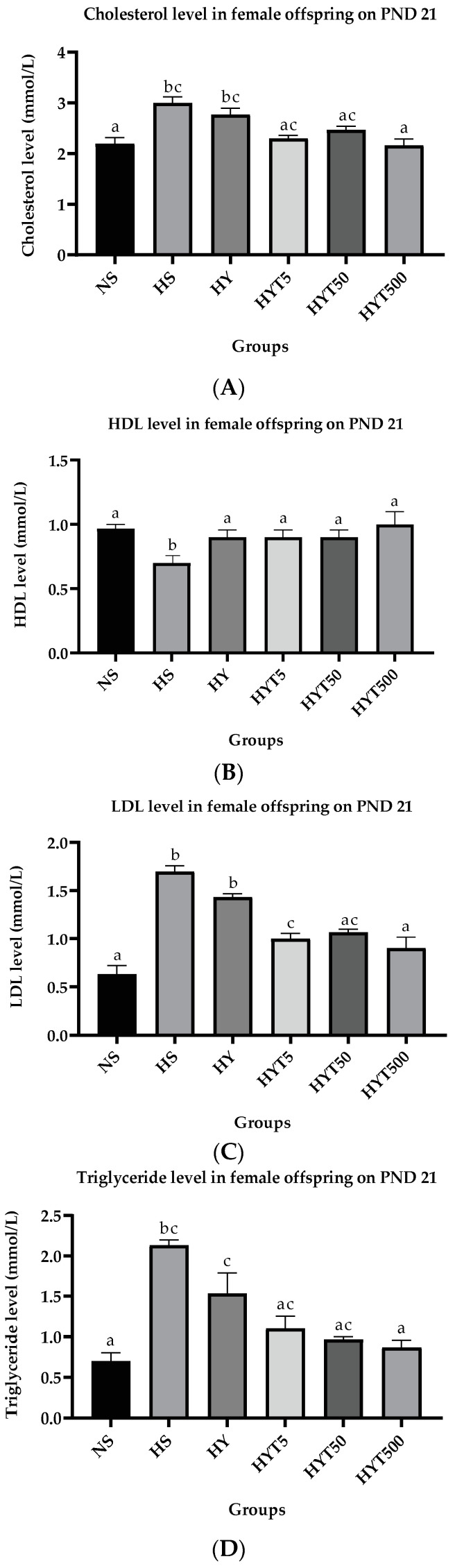
(**A**). Cholesterol level in female offspring on PND 21. (**B**). HDL level in female offspring on PND 21. (**C**). LDL level in female offspring on PND 21. (**D**). Triglycerides level in female offspring on PND 21. NS: normal chow and saline; HS: HFD and saline; HY: HFD and yoghurt; HYT5: HFD and 5 mg/kg of *E. tapos* yoghurt; HYT50: HFD and 50 mg/kg of *E. tapos* yoghurt; HYT500: HFD and 500 mg/kg of *E. tapos* yoghurt. Values are expressed as mean ± SEM. Different letters indicate a significant difference at *p* < 0.05.

Figure 6A–D shows the plasma concentration of cholesterol, HDL, LDL, and triglycerides. As shown in Figure 6A, total cholesterol of female offspring in HS and HY is significantly high (*p* < 0.05) compared to NS. There is no significant difference (*p* > 0.05) between HS, HY, HYT5, and HYT50. The total cholesterol of female offspring in HYT500 is significantly decreased (*p* < 0.05) compared to HS and HY, with a similar mean value as in the NS group. As demonstrated in Figure 6B, the HDL level in plasma is significantly reduced (*p* < 0.05) in HS compared to NS. Meanwhile, the HDL in HY, HYT5, HYT50, and HYT500 is significantly high (*p* < 0.05) compared to HS. There is no significant difference (*p* > 0.05) in the plasma HDL level of female offspring in HY, HYT5, HYT50, and HYT500 compared to NS. However, the mean value for HYT500 for plasma HDL concentration is similar to NS group. As shown in Figure 6C, the plasma LDL level in female offspring in HS and HY groups is significantly high (*p* < 0.05) compared to NS. Meanwhile, the plasma LDL level of HYT5, HYT50, and HYT500 is significantly low (*p* < 0.05) compared to HS and HY, but there is no significant difference (*p* > 0.05) in HYT5, and HYT50 for the plasma LDL level, while HYT50 and HYT500 show no significant difference (*p* > 0.05) compared to NS for plasma LDL concentration. Similarly, the triglycerides content in plasma of female offspring in HS and HY groups is significantly high (*p* < 0.05) compared to NS. In this, HYT5 and HYT50 show gradual reduction in plasma triglycerides, however, no significant difference (*p* > 0.05) in HYT5 and HYT500 compared to HS, HY, and NS. Meanwhile, the triglycerides content in female offspring’s plasma of HYT500 is significantly decreased (*p* < 0.05) compared to HS and HY, with a similar mean value as in the NS group as demonstrated in Figure 6D.

#### 3.1.8. *E. tapos* Yoghurt Prevents Fat Concentration (%) in Female Offspring

Table 1 shows the fat percentage in visceral, RpWAT, BAT, and gonadal of female offspring on PND 21. The result shows that the visceral, RpWAT, BAT, and gonadal fat percentage in female offspring of HS and HY groups is significantly higher (*p* < 0.05) compared to NS. In this, significant reduction (*p* < 0.05) in female offspring of HYT5, HYT50, and HYT500 for visceral, RpWAT, BAT, and gonadal fat percentage is seen compared to HS and HY groups. There is no significant difference (*p* > 0.05) in fat percentage of visceral, RpWAT, BAT, and gonadal fat of female offspring in HYT5, HYT50, and HYT500 compared to NS. However, the mean value of HYT500 for visceral, RpWAT, BAT, and gonadal fat percentage is comparable to NS.

#### 3.1.9. *E. tapos* Yoghurt Decreases Insulin Level in Female Offspring

**Figure 7:**
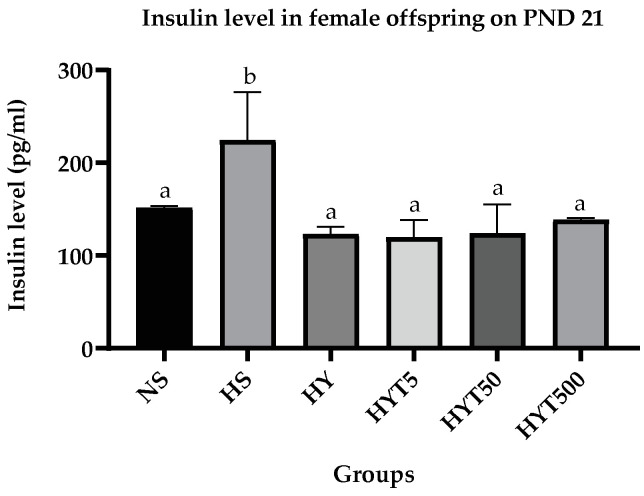
Plasma insulin level of female offspring on PND 21. NS: normal chow and saline; HS: HFD and saline; HY: HFD and yoghurt; HYT5: HFD and 5 mg/kg of *E. tapos* yoghurt; HYT50: HFD and 50 mg/kg of *E. tapos* yoghurt; HYT500: HFD and 500 mg/kg of *E. tapos* yoghurt. Values are expressed as mean ± SEM. Different letters indicate a significant difference at *p* < 0.05.

Figure 7 shows the insulin concentration on plasma of female offspring on PND 21. The data show that the insulin level of female offspring in HS is significantly high (*p* < 0.05) compared to NS. The plasma insulin concentration of HY, HYT5, HYT50, and HYT500 is significantly low (*p* < 0.05) compared to HS and there is no significant difference (*p* > 0.05) in plasma insulin concentration between HY, HYT5, HYT50, and HYT500 compared to NS. However, the mean value of HYT500 for the plasma insulin level is identical to NS.

### 3.2. Antioxidant Status

#### 3.2.1. *E. tapos* Yoghurt Increases FRAP Level in Serum and Hypothalamus in Female Offspring

**Figure 8:**
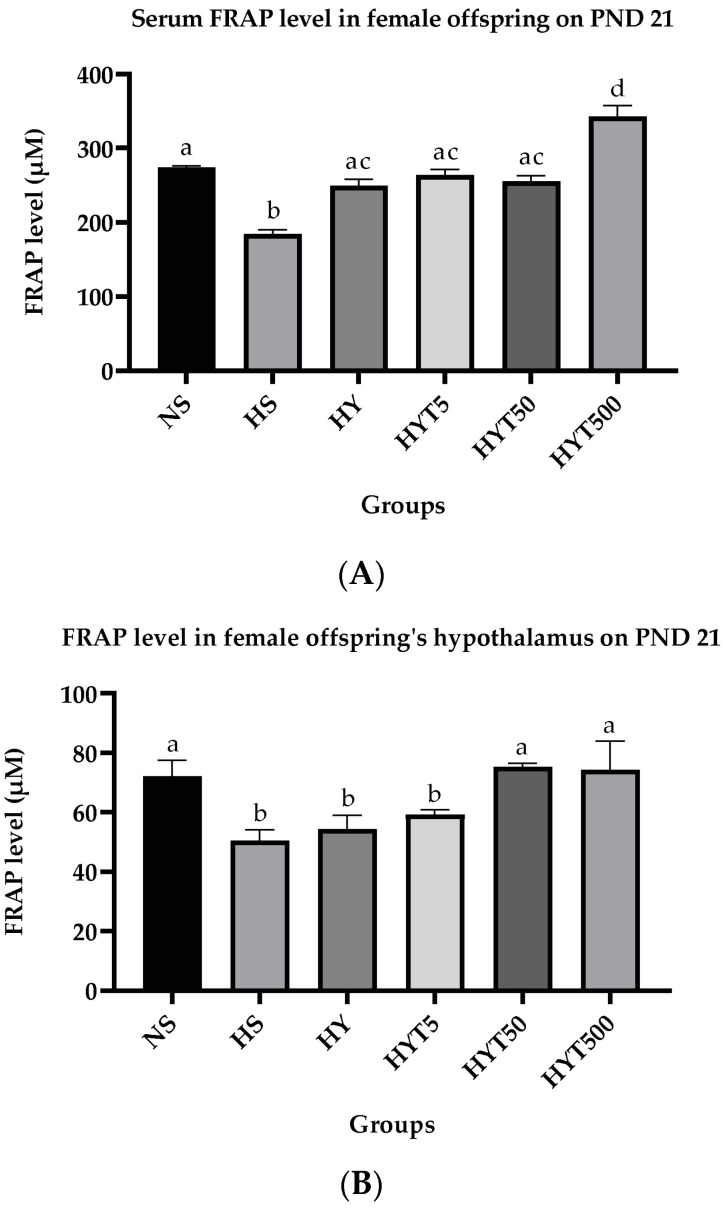
(**A**). Serum FRAP level in female offspring on PND 21. (**B**). FRAP level in hypothalamus of female offspring on PND 21. NS: normal chow and saline; HS: HFD and saline; HY: HFD and yoghurt; HYT5: HFD and 5 mg/kg of *E. tapos* yoghurt; HYT50: HFD and 50 mg/kg of *E. tapos* yoghurt; HYT500: HFD and 500 mg/kg of *E. tapos* yoghurt. Values are expressed as mean ± SEM. Different letters indicate a significant difference at *p* < 0.05.

Figure 8A,B shows the FRAP level in the serum and hypothalamus of female offspring on PND 21. In serum, the FRAP level in female offspring of HS is significantly low (*p* < 0.05) compared to NS. The serum level in HY, HYT5, and HYT50 is significantly high (*p* < 0.05) compared to HS and shows no significant difference (*p* > 0.05) compared to NS. Meanwhile, the serum FRAP level of female offspring in HYT500 is significantly high (*p* < 0.05) compared to NS, HS, HY, HYT5, and HYT50 on PND 21. As shown in Figure 8B, the FRAP level in the hypothalamus of female offspring in HS, HY, and HYT5 is significantly low (*p* < 0.05) compared to NS. Meanwhile, the FRAP level in the hypothalamus of female offspring in HYT50 and HYT500 is significantly high (*p* < 0.05) compared to HS, HY, and HYT5. There is no significant difference (*p* > 0.05) in the hypothalamic FRAP level in HYT50 and HYT500 compared to NS.

#### 3.2.2. *E. tapos* Yoghurt Increases the GSH Level in Serum and Hypothalamus in Female Offspring

**Figure 9:**
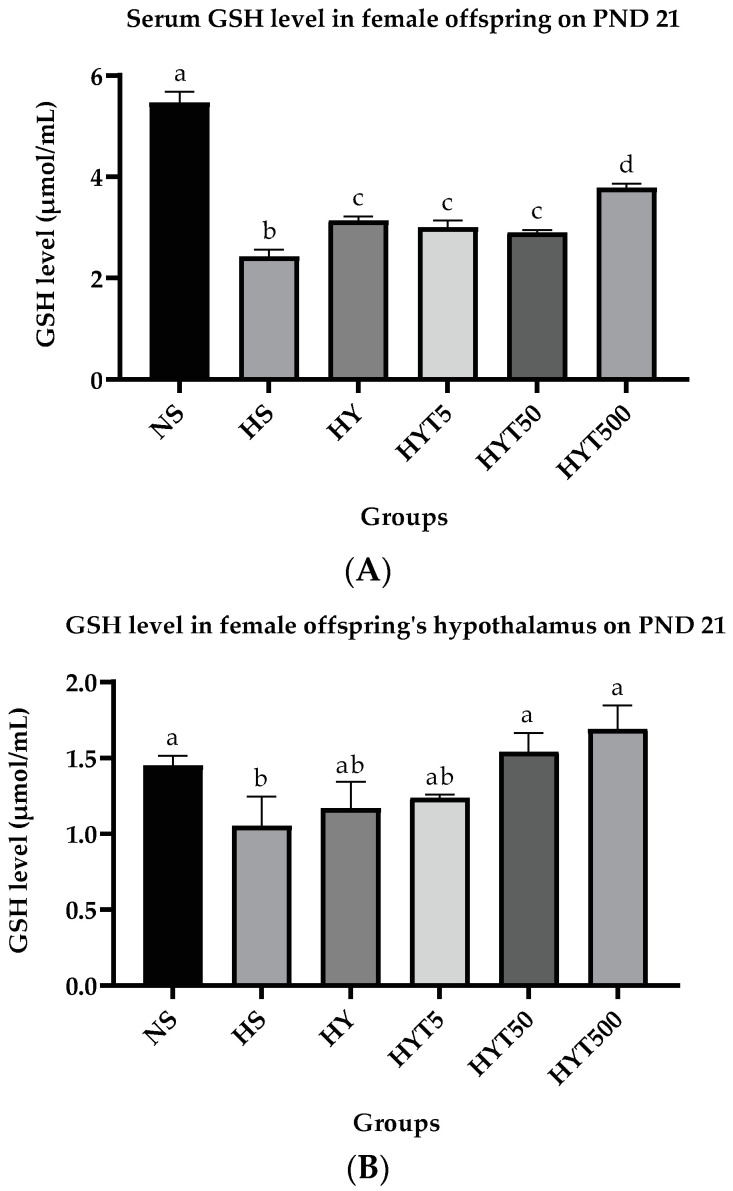
(**A**). Serum GSH level in female offspring on PND 21. (**B**). GSH level in hypothalamus of female offspring on PND 21. NS: normal chow and saline; HS: HFD and saline; HY: HFD and yoghurt; HYT5: HFD and 5 mg/kg of *E. tapos* yoghurt; HYT50: HFD and 50 mg/kg of *E. tapos* yoghurt; HYT500: HFD and 500 mg/kg of *E. tapos* yoghurt. Values are expressed as mean ± SEM. Different letters indicate a significant difference at *p* < 0.05.

Figure 9A,B shows the GSH level in the serum and hypothalamus of female offspring on PND 21. As demonstrated in Figure 9A, the serum GSH level in female offspring of HS, HY, HYT5, HYT50, and HYT500 is significantly low (*p* < 0.05) compared to NS. A significant increase (*p* < 0.05) in the serum GSH level in HY, HYT5, and HYT50 compared to HS is recorded on PND 21, while the serum GSH level of HYT500 is significantly high (*p* < 0.05) compared to HS, HY, HYT5, and HYT500 on PND 21. As shown in Figure 9B, the GSH level in the hypothalamus of female offspring in HS is significantly low (*p* < 0.05) compared to NS. There is a slight increase of the GSH level in the hypothalamus of HY and HYT5, however, there is no significant difference in the GSH level in the hypothalamus of HY and HYT5 compared to HS and NS. Meanwhile, the hypothalamic GSH level in HYT50 and HYT500 is significantly high (*p* < 0.05) compared to HS, with a similar mean value to the NS group.

## 4. Discussion

Human and animal study proves that excessive weight gain during pregnancy has a detrimental effect on the child’s metabolic profile [5]. Such change is a key factor for childhood obesity and poor cognitive performance. One of the common pathological changes observed in such condition includes dysregulation of glucose/insulin homoeostasis in the child [32]. In this context, consumption of HFD is known to cause maternal obesity and is positively associated with poor neurodevelopmental progress in the offspring [33]. Relevant to this statement, HFD is used to induce obesity in dams and the offspring in this study. In fact, gender specific studies show that females are more prone to anxiety disorders [34] and possess a high level of fat mass compared to males [35]. This makes the female offspring more susceptible to metabolic dysfunction and high potential to develop obesity [36]. Thus, in the current experimental study, we investigated the remodulation effect of *E. tapos* yoghurt on the metabolic profile of maternal obesity induced cognitive dysfunction and anxiety-like behavior in female offspring. This study specifically focuses on metabolic parameter changes, such as in FBG, insulin, lipid profile, fat concentration in different fat tissue, and how it affects the antioxidants status as well as cognitive function and thigmotactic behavior. The key findings from this study prove that the *E. tapos* yoghurt intervention during gestation in obese dams successfully reversed the maternal obesity inherited cognitive decline, anxiety-like behavior, and antioxidants status in the serum and hypothalamus of the female offspring.

Animal studies have demonstrated that excessive intake of HFD by dams during pregnancy leads to a high level of adiposity and cholesterol on PND 21 [37,38]. Similarly, a meta-regression study shows that female offspring of HFD fed dams shows persistent increase of triglycerides, FBG level, and insulin concentration [39]. Dysregulation of glucose homeostasis in obesegenic exposed offspring may appear as insulin resistance during their adolescence [40,41]. Thus, the high BMI and lee index of the offspring after weaning mimics the maternal obesity inherited obesogenic offspring rat model [42,43]. Consistent with this previous study, the BMI and lee index of female offspring of HFD supplemented dams is significantly heavier compared to the NS group. In fact, the female offspring of the HS group shows a significant high level of FBG, insulin, fat percentage, cholesterol, and triglycerides level. This signifies the successful establishment of the female F1 obesogenic rat model in this study. This further supports the theory that maternal overnutrition during the gestational period could affect lipid metabolism, causing an abnormal increase of fatty acid synthase in the offspring. This may result in dysregulation of the lipid profile which may always manifest as high levels of cholesterol and triglycerides in the plasma [44]. Another plausible explanation for this could be the increased level of transplacental lipid transport in maternal obesity accompanied by suppressed fatty acid protein and fatty acid binding proteins across the placenta. This may stimulate the lipid esterification in the placenta, causing an excessive level of lipid droplet to form, eventually leading to in utero insult that may cause the inheritance of obesity due to metabolic dysregulation [45].

Anyhow, female offspring of *E. tapos* yoghurt treated dams show similar fat percentage, cholesterol, LDL, and triglycerides level as in the NS group. This could be due to the high concentration of flavonoids and phenols in the *E. tapos* extract. Phytochemical assessment on *E. tapos* seed further proves that the high composition of flavonoids could potentially stop the action of α-amylase, α-glucosidase, and pancreatic lipase [17]. In this context, inhibition of α-amylase and α-glucosidase prevents the digestion of carbohydrates and slows down the increase in the glucose level in the blood. This is because oligosaccharides are formed through the binding of α-amylase to the glycosidic linkage from carbohydrates, which will then will bind to α-glucosidase to form monosaccharide sugars. Thus, inhibiting the activity of α-amylase and α-glucosidase will evince as low sugar levels in the blood [46]. Likewise, inhibition of pancreatic lipase activity intercepts the formation of monoglycerides and fatty acids by preventing the triglycerides hydrolysis. This could reduce the absorption of dietary fats up to one third [47]. Therefore, the levels of triglycerides and cholesterol will stay low in the bloodstream. In the meantime, the high concentration of unsaturated fatty acids in *E. tapos* seed [17] favors the liver’s activity as well. In that regard, the liver tends to transform unsaturated fatty acids into ketone bodies instead of LDL, eventually causing the appearance of LDL to decrease [48] and HDL to increase in the plasma [49]. In addition, high content of flavonoids in *E. tapos* is capable of suppressing the activity of PPARy and stimulates the activity of lipolysis, thereby inhibiting adipogenesis [50]. This could be the probable reason for an identical fat percentage in female offspring of HYT5, HYT50, and HYT500 as in the NS group. Hence, all these changes induced by *E. tapos* yoghurt in obese dam’s female offspring give a conceivable reason for low BMI, lee index, and waist circumference in HYT5, HYT50, and HYT500 compared to the HS group.

In addition, offspring of HFD induced maternal obesity tend to suppress the level of endogenous antioxidants. A study conducted by Moraes-Souza et al., 2021 demonstrated that maternal intake of HFD induced oxidative stress on the fetus, subsequently initiating abnormal expressions of the gene associated with antioxidant defense of the offspring, causing the level of antioxidants to decrease [51]. In parallel to this claim, the levels of FRAP and GSH were significantly lower in the female offspring of the HS group in the serum and hypothalamus, suggesting the presence of excessive level oxidative stress in the female offspring. A low level of antioxidant defense, together with lipid rich constitution, makes the brain susceptible to redox imbalance. This redox imbalance interferes in the neuronal transmission, disrupts the integrity of the membrane, eventually leading to neuronal apoptosis. This serves as one of the primary precursors for high anxiety status [52] and memory impairment [53]. This gives a logical justification for the anxiety-like behavior exhibited by female offspring belonging to the HS group in this study. On the contrary, the female offspring of *E. tapos* administered obese dams show a gradual increase of FRAP and GSH in the serum and hypothalamus, with a similar recognition index for novel objects and place recognition. This is mainly due to the existence of strong antioxidants in *E. tapos* seed as observed by Tisadondilok et al., 2018 in their study [19]. Apace with their results, a high level of the FRAP value in the serum and hypothalamus in our study is the evidence that *E. tapos* yoghurt is a strong antioxidant. This is because antioxidants could exhibit protective effects against oxidative stress and block the maternal obesity induced anxiety phenotype in the offspring. Simultaneously, antioxidants prevent the shortening of dendrites, and reduction of gamma-aminobutyric acid subunit receptors in the hippocampus, thereby inhibiting the development of anxiety-like behavior in the offspring [54]. A similar mechanism explains that the cause for maternal subjects exposed to antioxidants exhibits more protective effects against memory decline and hippocampal atrophy in the offspring [55]. Hence, this explains the underlying mechanism for the high level of the recognition index demonstrated by female offspring in HYT5, HYT50, and HYT500 compared to the HS group. The *E. tapos* intervene groups show more prominent and consistent positive outcome throughout all the parameters assessed in this study. The integration of natural extract (*E. tapos*) into yoghurt is proven to stimulate probiotic growth [56] and integration of *E. tapos* into yoghurt prevents the placental barrier crossing of HFD induced oxidative stress from maternal into the fetus [57]. This further inhibit the fetus to inherit the metabolic dysregulation as in the maternal obese subjects. Henceforth, this study shows beyond a doubt that *E. tapos* yoghurt is a beneficial supplement that can re-modulate the metabolic profile of maternal obesity induced cognitive dysfunction and anxiety-like behavior in female offspring.

## 5. Conclusions

In conclusion, this experimental study demonstrates that HFD supplementation during pregnancy is the root cause for metabolic profile dysregulation in the female offspring. However, the supplementation of *E. tapos* yoghurt reverses the action of HFD induced maternal obesity inherited metabolic dysregulation, cognitive performance, and anxiety-like behavior in female offspring at the dose of 500 mg/kg/day.

## Figures and Tables

**Table 1:** Percentage of fat in visceral, BAT, gonadal, and RpWAT of female offspring on PND 21.

Group/Organ	BAT	RpWAT	Gonadal	Visceral
NS	0.11 ± 0.03 ^a^	0.20 ± 0.08 ^a^	0.25 ± 0.12 ^a^	0.63 ± 0.07 ^a^
HS	0.25 ± 0.02 ^b^	1.02 ± 0.23 ^b^	1.52 ± 0.65 ^b^	1.17 ± 0.12 ^b^
HY	0.22 ± 0.04 ^b^	0.85 ± 0.15 ^b^	1.08 ± 0.36 ^b^	0.81 ± 0.05 ^ab^
HYT5	0.15 ± 0.02 ^a^	0.27 ± 0.06 ^a^	0.35 ± 0.04 ^a^	0.66 ± 0.06 ^a^
HYT50	0.14 ± 0.03 ^a^	0.26 ± 0.02 ^a^	0.31 ± 0.03 ^a^	0.59 ± 0.07 ^a^
HYT500	0.10 ± 0.02 ^a^	0.22 ± 0.08 ^a^	0.28 ± 0.09 ^a^	0.65 ± 0.04 ^a^

NS: normal chow and saline; HS: HFD and saline; HY: HFD and yoghurt; HYT5: HFD and 5 mg/kg of *E. tapos* yoghurt; HYT50: HFD and 50 mg/kg of *E. tapos* yoghurt; HYT500: HFD and 500 mg/kg of *E. tapos* yoghurt; BAT: brown adipose tissue; RpWAT: retroperitoneal white adipose tissue. Values are expressed as mean ± SEM. Different letters indicates a significant difference at *p* < 0.05.

## Data Availability

The data are available from the corresponding author.
